# Supplementary material for: Macrophage-Derived Factors with the Potential to Contribute to Pathogenicity of HIV-1 and HIV-2: Role of CCL-2/MCP-1
Source: Viruses. 2023 Oct 27;15(11):2160. doi: 10.3390/v15112160 (PMC10674259; doi:10.3390/v15112160)
Supplement: Supplementary file 1 [file viruses-15-02160-s001.zip › viruses-2506174-supplementary.pdf]

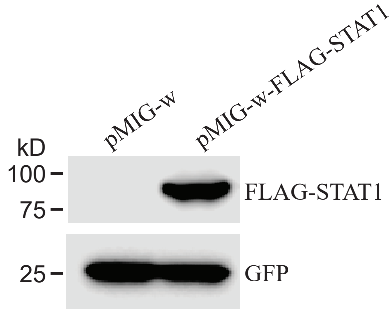

Figure S1. Expression of STAT1 in the transfected 293T cells. 293T cells were transfected with the empty pMIG-w or pMIG-w-FLAG-STAT1 plasmid. Cells were extracted 48 hours following the transfection. The expression of FLAG-STAT1 in the cell lysates was determined by Western blot using anti-FLAG antibody from Sigma (M2, Cata# F3165). GFP was used as a control for expression of the STAT1-IRES-GFP cassette.

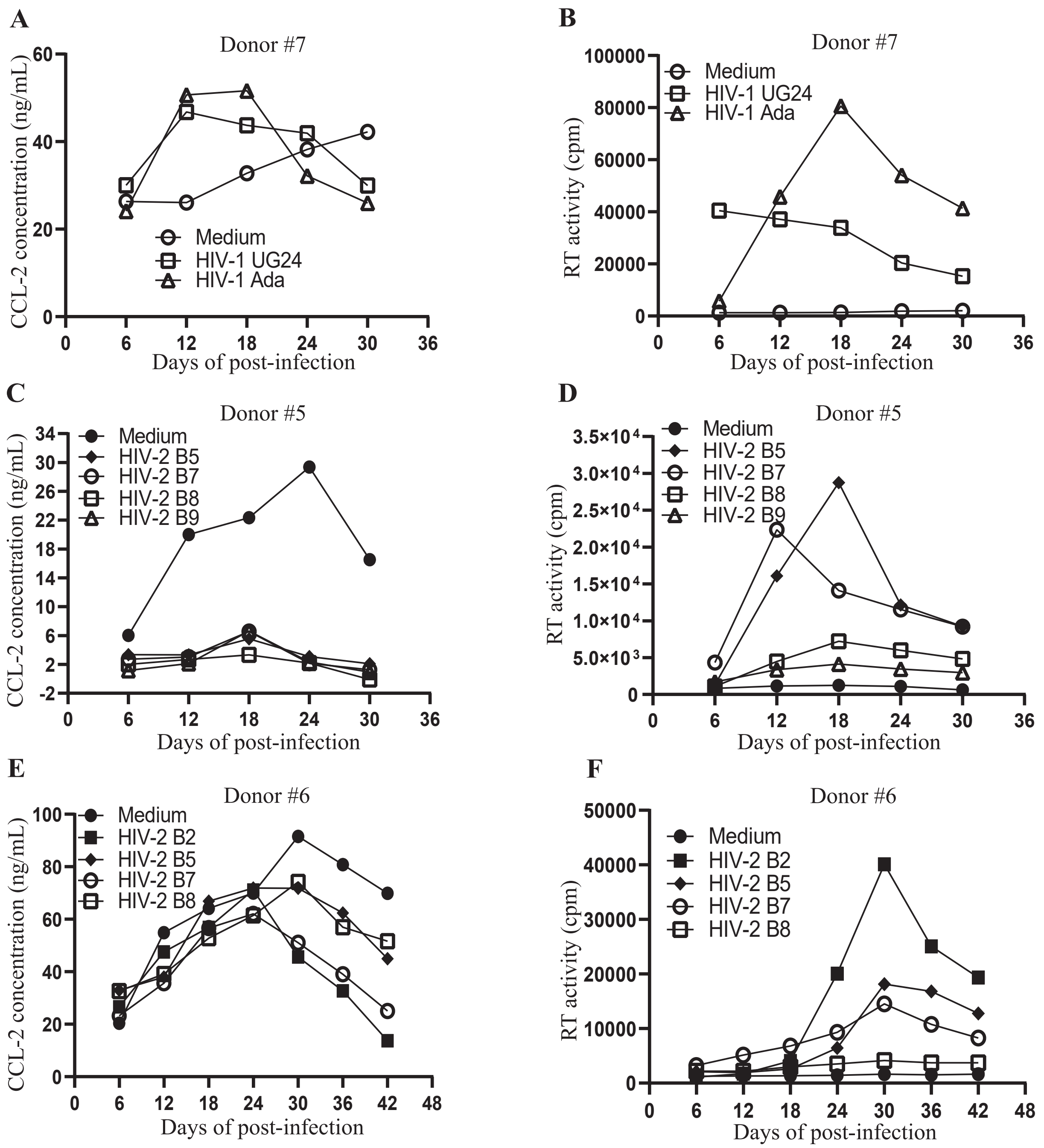

Figure S2. Virus replication (B, D, F) and CCL2 expression (A, C, E) in MDM infected with HIV-2 (C- F) or HIV-1 (A, B) isolates. MDM were differentiated from primary monocytes islolated from donors #5 (C, D), #6 (E, F) and #7 (A, B). The CCL2 expression levels of MDM following the infection were determined by ELISA at the indicated time points. Virus replication was monitored by measuring RT activity in the supernatant harvested at the indicated time points.

Table S1. Virus replication and CCL2 induction in MDM infected with HIV-1 isolates

Donor #8	HIV-1 UG24		HIV-1BCFO3		HIV-1 Ada		HIV-1 Bal	
	RT (fold) [1]	CCL2 (fold) [1]	RT (fold)	CCL2 (fold)	RT (fold)	CCL2 (fold)	RT (fold)	CCL2 (fold)
Day 9	0.94	-0.31	14.81	1.96	1.42	1.58	36.57	1.98
Day 15	2.10	0.06	46.30	0.77	108.67	1.12	93.97	5.28
Day 21	5.40	-0.42	35.80	0.71	85.29	0.94	77.86	3.56
Day 27	3.83	-0.71	51.47	-0.26	80.57	0.05	69.54	0.86
Day 33	2.63	-0.70	22.03	-0.39	28.84	-0.60	65.41	0.54

[1] Fold change was calculated using the formula: (RT/CCL2 of HIV-1-infected MDM – RT/CCL2 of uninfected MDM) ÷ (RT/CCL2 of uninfected MDM)

Table S2. Virus replication and CCL2 induction in MDM infected with HIV-1 isolates

Donor #7	HIV-1 UG24		HIV-1 Ada	
	RT (fold) [1]	CCL2 (fold) [1]	RT (fold)	CCL2 (fold)
Day 6	29.43	0.14	3.79	-0.08
Day 12	28.98	0.80	36.80	0.94
Day 18	21.88	0.34	52.50	0.58
Day 24	10.62	0.10	27.94	-0.16
Day 30	8.66	-0.29	23.24	-0.38

[1] Fold change was calculated using the formula: (RT/CCL2 of HIV-1-infected MDM – RT/CCL2 of uninfected MDM) ÷ (RT/CCL2 of uninfected MDM)

Table S3. Virus replication and CCL2 induction in MDM infected with HIV-2 isolates

Donor #8	HIV-2 B2		HIV-2 B3		HIV-2 B4		HIV-2 B5		HIV-2 B7		HIV-2 B8		HIV-2 B9	
	RT (fold) [1]	CCL2 (fold) [1]	RT (fold)	CCL2 (fold)	RT (fold)	CCL2 (fold)	RT (fold)	CCL2 (fold)	RT (fold)	CCL2 (fold)	RT (fold)	CCL2 (fold)	RT (fold)	CCL2 (fold)
Day 9	-0.16	-0.46	-0.01	0.12	0.63	-0.39	0.14	-0.44	0.17	-0.43	-0.25	-0.76	-0.04	0.54
Day 15	-0.29	-0.72	0.79	-0.59	9.23	-0.78	14.41	-0.73	2.04	-0.90	-0.32	-1.00	0.13	-0.56
Day 21	-0.25	-0.55	3.15	-0.49	9.35	-0.90	20.93	-0.82	3.18	-0.90	-0.08	-1.01	0.75	-0.29
Day 27	0.81	-0.24	10.52	-0.67	19.35	-0.96	34.50	-0.86	8.53	-0.93	-0.17	-1.01	1.14	-0.01
Day 33	0.77	0.15	12.35	-0.66	16.02	-0.94	24.15	-0.81	5.76	-0.91	-0.11	-1.01	0.94	-0.12

[1] Fold change was calculated using the formula: (RT/CCL2 of HIV-2-infected MDM – RT/CCL2 of uninfected MDM) ÷ (RT/CCL2 of uninfected MDM)

Table S4. Virus replication and CCL2 induction in MDM infected with HIV-2 isolates

Donor #5	HIV-2 B5		HIV-2 B7		HIV-2 B8		HIV-2 B9	
	RT (fold) [1]	CCL2 (fold) [1]	RT (fold)	CCL2 (fold)	RT (fold)	CCL2 (fold)	RT (fold)	CCL2 (fold)
Day 6	0.63	-0.44	4.30	-0.55	0.32	-0.67	1.07	-0.81
Day 12	13.04	-0.83	18.51	-0.85	2.90	-0.87	1.94	-0.89
Day 18	21.98	-0.75	10.27	-0.71	4.77	-0.85	2.29	-0.70
Day 24	10.17	-0.89	9.62	-0.93	4.52	-0.93	2.19	-0.92
Day 30	13.85	-0.87	13.80	-0.92	6.75	-1.01	3.77	-0.94

[1] Fold change was calculated using the formula: (RT/CCL2 of HIV-2-infected MDM – RT/CCL2 of uninfected MDM) ÷ (RT/CCL2 of uninfected MDM)

Table S5. Virus replication and CCL2 induction in MDM infected with HIV-2 isolates

Donor #6	HIV-2 B2		HIV-2 B5		HIV-2 B7		HIV-2 B8	
	RT (fold) [1]	CCL2 (fold) [1]	RT (fold)	CCL2 (fold)	RT (fold)	CCL2 (fold)	RT (fold)	CCL2 (fold)
Day 6	-0.12	0.31	0.68	0.62	1.59	0.14	0.75	0.60
Day 12	0.30	-0.13	0.46	-0.31	2.86	-0.35	0.65	-0.29
Day 18	1.95	-0.11	0.91	0.04	3.97	-0.12	1.17	-0.18
Day 24	12.89	0.02	3.47	0.03	5.47	-0.11	1.43	-0.12
Day 30	23.39	-0.50	10.02	-0.22	7.83	-0.44	1.51	-0.19
Day 36	15.59	-0.60	10.13	-0.23	6.13	-0.52	1.46	-0.29
Day 42	10.81	-0.80	6.79	-0.36	4.06	-0.64	1.27	-0.26

[1] Fold change was calculated using the formula: (RT/CCL2 of HIV-2-infected MDM – RT/CCL2 of uninfected MDM) ÷ (RT/CCL2 of uninfected MDM)

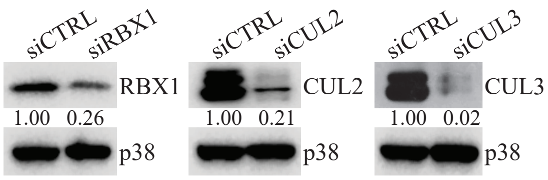

Figure S3. Knockdown of RBX1, CUL2 and CUL3 by siRNA. MDM were transfected with control siRNA (siCTRL), or siRNA targeting human RBX1 (siRBX1), CUL2 (siCUL2) or CUL3 (siCUL3). Cells were extracted 96 hours following the siRNA transfection. The levels of RBX1, CUL2 and CUL3 are determined by Western blotting. p38 was used as a loading control.
